# 

**Published:** 1816-02

**Authors:** 


### CORRESPONDENCE.

Dr. Curry's Communication, Mr. M'Leod on the Bulam Fever; Medico-Chirurgus; and a "curious Instance of Malformation," shall appear in our next Number.

